# Motor learning in developmental coordination disorder: behavioral and neuroimaging study

**DOI:** 10.3389/fnins.2023.1187790

**Published:** 2023-06-22

**Authors:** Emad Al-Yahya, Patrick Esser, Benjamin D. Weedon, Shawn Joshi, Yan-Ci Liu, Daniella N. Springett, Piergiorgio Salvan, Andy Meaney, Johnny Collett, Mario Inacio, Anne Delextrat, Steve Kemp, Tomas Ward, Hooshang Izadi, Heidi Johansen-Berg, Hasan Ayaz, Helen Dawes

**Affiliations:** ^1^School of Health Sciences, University of Nottingham, Nottingham, United Kingdom; ^2^School of Rehabilitation Sciences, University of Jordan, Amman, Jordan; ^3^Centre for Movement, Occupation and Rehabilitation Services, Oxford Brookes University, Oxford, United Kingdom; ^4^School of Biomedical Engineering, Science and Health Systems, Drexel University, Philadelphia, PA, United States; ^5^College of Medicine, Drexel University, Philadelphia, PA, United States; ^6^School and Graduate Institute of Physical Therapy, College of Medicine, National Taiwan University, Taipei City, Taiwan; ^7^Department for Health, University of Bath, Bath, United Kingdom; ^8^Wellcome Centre for Integrative Neuroimaging, Nuffield Department of Clinical Neurosciences, University of Oxford, Oxford, United Kingdom; ^9^Research Center in Sports Sciences, Health Sciences and Human Development, University of Maia, Porto, Portugal; ^10^Insight SFI Research Centre for Data Analytics, Dublin City University, Dublin, Ireland; ^11^School of Engineering, Computing and Mathematics, Oxford Brookes University, Oxford, United Kingdom; ^12^Department of Psychological and Brain Sciences, College of Arts and Sciences, Drexel University, Philadelphia, PA, United States; ^13^Drexel Solutions Institute, Drexel University, Philadelphia, PA, United States; ^14^Department of Family and Community Health, University of Pennsylvania, Philadelphia, PA, United States; ^15^Center for Injury Research and Prevention, Children’s Hospital of Philadelphia, Philadelphia, PA, United States; ^16^NIHR Exeter BRC, Medical School, University of Exeter, Exeter, United Kingdom

**Keywords:** developmental coordination disorder, motor control, prefrontal cortex, frontoparietal networks, fNIRS, MRI

## Abstract

Developmental coordination disorder (DCD) is characterized by motor learning deficits that are poorly understood within whole-body activities context. Here we present results of one of the largest non-randomized interventional trials combining brain imaging and motion capture techniques to examine motor skill acquisition and its underpinning mechanisms in adolescents with and without DCD. A total of 86 adolescents with low fitness levels (including 48 with DCD) were trained on a novel stepping task for a duration of 7 weeks. Motor performance during the stepping task was assessed under single and dual-task conditions. Concurrent cortical activation in the prefrontal cortex (PFC) was measured using functional near-infrared spectroscopy (fNIRS). Additionally, structural and functional magnetic resonance imaging (MRI) was conducted during a similar stepping task at the beginning of the trial. The results indicate that adolescents with DCD performed similarly to their peers with lower levels of fitness in the novel stepping task and demonstrated the ability to learn and improve motor performance. Both groups showed significant improvements in both tasks and under single- and dual-task conditions at post-intervention and follow-up compared to baseline. While both groups initially made more errors in the Stroop task under dual-task conditions, at follow-up, a significant difference between single- and dual-task conditions was observed only in the DCD group. Notably, differences in prefrontal activation patterns between the groups emerged at different time points and task conditions. Adolescents with DCD exhibited distinct prefrontal activation responses during the learning and performance of a motor task, particularly when complexity was increased by concurrent cognitive tasks. Furthermore, a relationship was observed between MRI brain structure and function measures and initial performance in the novel stepping task. Overall, these findings suggest that strategies that address task and environmental complexities, while simultaneously enhancing brain activity through a range of tasks, offer opportunities to increase the participation of adolescents with low fitness in physical activity and sports.

## Introduction

Developmental Coordination Disorder (DCD) is a chronic and prevalent neurodevelopmental disorder affecting approximately 5% of the population (Lingam et al., 2009). The disorder is characterized by significant delay in the acquisition of motor skills (i.e., learning) and impairment in the execution of coordinated movements (i.e., control) (World-Health-Organization [WHO], 2022). Whilst deficits in motor learning and control are typically evident from an early age, they are not associated with physical, intellectual, or sensory impairments, and do not progress with time (Blank et al., 2019; Arthur et al., 2021). Learning coordinated movements is critical during the development period (Logan et al., 2018), and has been associated with successful participation in many everyday actions, school work, and sports and leisure activities (Robinson et al., 2015). Indeed, the acquisition of new motor skills has been associated with positive health outcomes and trajectories including increased physical activity, improved health-related fitness, enhanced perceived competence, and healthier weight status (Robinson et al., 2015; Logan et al., 2018). However, it is important to note that DCD is a rather continuum of disorders wherein affected individuals fall at the low end of the normal distribution of motor skills (Gomez and Sirigu, 2015).

Several meta-analyses and systematic reviews have consistently emphasized that training motor skills leads to improved performance (Bo and Lee, 2013; Morgan et al., 2013; Yu et al., 2018). Strength and conditioning interventions have also been shown to enhance performance in different domains (Robinson et al., 2015; Logan et al., 2018; Collins et al., 2019). However, in children with DCD the evidence is mixed, with variability in the speed that skills are learnt, the retention effects on motor performance, and the overall movement competence gained (Bo and Lee, 2013; Adams et al., 2014; Yu et al., 2018). The difficulty with developing successful interventions for children with DCD likely stems from a lack of understanding of the mechanisms underpinning motor skills learning in them (Collins et al., 2019; Subara-Zukic et al., 2022).

Lab based research, mainly in hand and eye movements, suggested that children with DCD exhibit a selective tendency to utilize feedback-driven control and/or learning strategies, whereby online sensory cues are increasingly sampled at the expense of internal action models (Steenbergen et al., 2020; Arthur et al., 2021). This selective deficit in the generation or utilization of internal models for feed-forward planning has been suggested to underpin movement impairments although much is unknown in ecologically valid tasks (Adams et al., 2014; Wilson et al., 2017). Converging evidence supports the deficits in the predictive control of movements hypothesis, as poor anticipatory planning was a common denominator in studies. However, to date there is limited research investigating factors associated with the size and rate of improvements when learning fundamental movement skills incorporating functional whole-body activities (Subara-Zukic et al., 2022).

Research findings also suggest that motor deficits in children with DCD are most evident under conditions of increased task complexity (Adams et al., 2014; Steenbergen et al., 2020; Jelsma et al., 2021). Under such challenging situations, motor difficulties have been attributed to increased demands on attentional and cognitive resources when performing novel tasks. This later notion is supported by both experimental brain imaging studies and investigations into training-induced plasticity in individuals with DCD (Wilson et al., 2017; Izadi-Najafabadi et al., 2020). Consistent findings emphasize the role of frontoparietal networks in responding to the overload of attentional and cognitive resources during complex task performance, motor learning and movement automatization. However, previous studies have to date focused on hand movements (Fuelscher et al., 2018), did not include response to physical rehabilitation intervention (Izadi-Najafabadi et al., 2022), and their findings could not be generalized to learning and performance of whole body and/or sporting activities (Rivilis et al., 2011; Biotteau et al., 2017). Indeed, there is a crucial need to further investigate and understand performance in challenging situations that involve duals tasks, tasks requiring precision, or those performed under time constraints. By examining how individuals with DCD navigate and perform in these challenging scenarios, we can gain a deeper understanding of the underlying mechanisms and limitations of motor control and learning, thus paving the way for more targeted interventions.

In this study, we combined brain imaging and motion capture techniques to examine motor skill acquisition and its underpinning mechanisms in young people with DCD. We hypothesized that adolescents with lower motor control, when performing a novel rhythmic stepping task, would exhibit differences in motor performance associated with brain activation patterns in terms of level of activation and/or brain areas involved. We also expected to observe variations in structural brain measures between two groups of adolescents with similar fitness levels but differing motor performance characteristics, both at baseline and in response to training. Furthermore, we hypothesized that adolescents with the lowest skills would acquire motor skill competence in a distinct manner, with their movement quality remaining lower compared to the other group. We anticipated that the DCD group would demonstrate a more controlled and less automated approach to task performance, as indicated by greater cortical brain activation during single task conditions, and their performance would be more disrupted during dual tasking, even after training. Our working hypothesis is that individuals with DCD, who may have deficits in adapting or implementing internal sensorimotor models of action, will demonstrate asynchrony between rhythmic cuing and movement compared to individuals without DCD. Additionally, we expected that a tendency to rely on performance errors would affect the ability to make instantaneous adjustments in movement, rendering sensory prediction errors ineffective in updating the internal model and thereby disrupting movement automatization. Finally, this study aimed to assess the feasibility of delivering an intervention within a school setting and to estimate parameters that would inform a larger and more substantial trial.

## Materials and methods

A detailed protocol was published previously (Esser et al., 2019). In brief, the study was a trial comparing motor learning and performance between adolescents (Sawyer et al., 2018) with and without DCD. The study protocol was reviewed and approved by local institutional review board (UREC 161033) and registered on clinicaltrials.gov (NCT03150784). All procedures were carried out in accordance with the latest guidelines and regulations of the Declaration of Helsinki. All potentially eligible participants were provided with details of the study via parental evenings and written information sheets. Participant representatives were involved in the design, conduct and dissemination of the study. All participants and parents/guardians provided informed consent prior to participation in this research study.

### Participants recruitment and allocation

Potential participants were identified from a cohort of students in three mainstream schools in Oxfordshire, UK. Adolescents were screed using the Movement Assessment Battery for Children-2nd edition (MABC-2) (ACSM, 2013) and the shuttle-run test (Léger et al., 1988). Participants were eligible if they were: (1) in the lowest quartile of fitness, as per the shuttle-run test, (2) had normal intelligence as reported by teachers, and (3) had no contraindications to exercise. Participants were excluded if they have any of the following criteria: (1) behavioral, cognitive, or intellectual issues that would prevent safe participation; (2) contraindications to perform maximal exercise or physical training; (3) muscular or neurological degenerative conditions; and (4) surgery in the previous 6 months.

Interested adolescents with poor motor skill acquisition [MABC-2 scores ≤ 15th percentile (World-Health-Organization [WHO], 1992)] and teacher confirmed performance affecting daily functioning were allocated to the DCD group and those in the lower quartile of fitness without poor motor skill acquisition and execution (MABC-2 scores > 15th percentile) were allocated to the typically developing (TD) group.

### Procedures and measures

This study was repeated measures (pre, post, and follow-up) mixed within- and between-subjects design. Assessors were blinded to group allocation. The study comprised a 7 weeks intervention and a 12 weeks follow-up period.

### Demographics

Demographic and descriptive measures at baseline included Child Health Utility Questionnaire 9D (CHU9D) for quality of life (Stevens, 2012), Harter’s Self-Perception Profile for Children (HSPC), Physical Activity Questionnaire for Adolescents (PAQ-A) (Kowalksi et al., 1997), and body mass index (BMI).

### Motor and cognitive performance

A novel rhythmic motor task was utilized to assess participants’ motor performance. The task requires alternating stepping on an exercise step (approximately 20 cm high) at a fixed frequency of 0.5 Hz. Stepping was instructed via a visual cue that was displayed for 1.5 s (“LEFT” or “RIGHT” displayed on the corresponding side of the screen) followed by 0.5 s of a blank screen. Participants repeated 10 steps with each side. Participants were fitted with an inertial measurement unit (IMU, LPMS-B, Life Performance Research, Tokyo, Japan) to record tri-axial accelerometry of the center of mass (Esser et al., 2009). Linear acceleration was used to measure stepping frequency and step time variability. Stepping frequency adherence was estimated by relative power spectral density (PSD) at 0.5 Hz that reflects the proportion of spectral energy focused in the 0.5 Hz target stepping frequency (expressed as a percentage) (Skiadopoulos and Stergiou, 2020). Step time coefficient of variation (CoV) was used to measure step time variability.

The motor task was performed either under single- or dual-task conditions. During the dual-task condition, participants performed the rhythmic stepping simultaneously with an auditory Stroop task (Morgan and Brandt, 1989). The auditory Stroop task involved listening to the words “high” and “low” at a high and a low pitch and quickly specify the pitch of the word as accurate as possible (Plummer-D’Amato et al., 2012). The auditory task was also repeated as a single-task while standing and performance in it was quantified by counting errors, expressed as the percentage of wrong answers in both single- and dual-task conditions. Each of the three tasks (i.e., motor alone, auditory alone, and motor and auditory concurrently) was completed three times for a total of nine blocks in a pseudo-random order.

### Measuring prefrontal cortex activation with fNIRS

**FIGURE 1:**
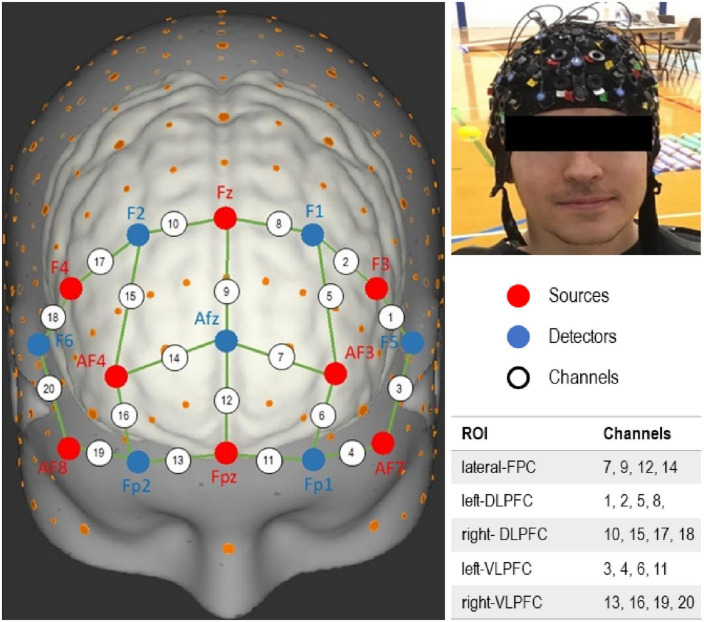
Function near-infrared spectroscopy (fNIRS) channels layout. Channels encompassing different regions of interest (ROI) within the prefrontal cortex (PFC) are identified.

A 20-channel, portable functional near infrared spectroscopy (fNIRS) device (NIRx Medical Technologies, NY, USA) was used to measure prefrontal cortex activation (PFC) during different tasks performance and at the three time points. Details on fNIRS data acquiring, processing, and analysis have been recently described (Joshi et al., 2022). For this analysis, we have identified a network of five regions of interests within the PFC; left and right dorsolateral PFC (DLPFC), left and right ventrolateral PFC (VLPFC), and lateral frontopolar cortex (FPC) (Figure 1). A 3D digitizing tool guided the selection of optodes positions (Zimeo Morais et al., 2018).

In brief, attenuation changes in raw light intensity were transformed to relative concentration changes in oxygenated hemoglobin (Oxy-Hb) using the modified Beer-Lambert law (Delpy et al., 1988). Data were then filtered to remove high frequency noise, cardiovascular contamination, and signal drift using an autoregressive model (Barker et al., 2013). Motion artifacts were resolved by a baseline correction algorithm (Santosa et al., 2018) followed by a wavelet filter (Molavi and Dumont, 2012). Furthermore, relative optical density data were visually inspected and those with insufficient quality were removed (Menant et al., 2020). We then conducted a feature extraction that involved the block average of the tasks per time and individual. Then, the average of relative changes in Oxy-Hb concentration of combined blocks was calculated for each task, region, and utilized for subsequent statistical analyses.

### Magnetic resonance imaging

Participants enrolled in the study were invited to take part in an MRI sub-study. Participants showing MR contraindications (e.g., braces, claustrophobia, surgical implants) were excluded. MRI-scans were carried out at baseline and post intervention in the Wellcome Centre for Integrative Neuroimaging (WIN), University of Oxford, using a 3T Siemens Magnetom Prisma (Erlangen, Germany) scanner with a 32-channel head coil. The MR-protocol comprised both functional and structural sequences. During the task condition participants were instructed to tap their feet alternatingly at a fixed frequency of 0.5 Hz for 30 s whereby a stimulus (LEFT/RIGHT) was visible for 1.5 s followed by a 0.5 s blank screen, programmed in Presentation (NBS, USA). Reciprocal feet movements were restricted to one degree of freedom (extension-flexion at the ankle joint) and measured via a tailored MR-safe device linked to potentiometers which recorded the positional angle in a customized LabVIEW program (National Instruments, Ireland).

Detailed MRI data acquisition and analysis is described in Supplementary material. In brief, MRI data were analyzed using tools from FMRIB Software Library (FSL) (Jenkinson et al., 2012). Baseline cross-sectional analyses were carried out while adjusting for age and gender. Longitudinal analyses were carried out on subject paired-differences (post-minus-pre) while adjusting only for gender. Voxel wise statistical testing was carried out through FSL randomize tool for non-parametric permutation inference. Statistical significance was assessed after 10,000 permutations and family-wise-error (FWE)-correction. Threshold-free cluster enhancement (TFCE) with 2D optimization carried out for trace-based spatial analyses of diffusion tensor imaging data; 3D TFCE was carried out otherwise. All FWE-corrected *P* < 0.05 were considered significant. Where a significant result was found, a subject-average value was extracted from the significant cluster and plotted to visualize the underlying data using scatter and/or box plots.

### Intervention

Both groups participated in an exercise intervention program that was delivered twice a week for 7 weeks. Exercise sessions were delivered at the participant’s school. Each 60 min session consisted of an initial warm up, moderate to vigorous intensity physical activity (50–85% maximum heart rate), and individualized stepping training protocol (Esser et al., 2019). Aerobic exercise could induce brain plasticity in early to middle adulthood, even within a relatively short timeframe of 6 weeks (Thomas et al., 2016). There is also a general consensus that the developing brain is more responsive to experiences compared to the adult brain (Kolb et al., 2017). Therefore, it is plausible that a behavioral intervention lasting seven weeks could potentially induce training-dependent brain plasticity in adolescents.

### Statistical analysis

Statistical analysis was performed using IBM-SPSS 28.0 (IBM SPSS, Inc., Armonk, NY, United States). At first, we performed descriptive statistical analysis on demographics. For behavioral data, mixed-design analysis of variance (ANOVA) was conducted separately for each of the performance variables; step time variability, PSD at 0.5 Hz, and percentage of errors in auditory Stroop performance. Time and Task were set as the independent within-subjects variables, and participant’s Group as the between subject factor.

Similarly, for each region of interest, fNIRS data from all participants were analyzed by conducting a mixed-design ANOVA with Time and Task as the independent within-subjects variables while participant’s Group was the between-subjects factor. For all statistical tests, alpha level was set at 0.05 *a priori* and SPSS-generated Bonferroni adjusted *P*-values are quoted.

## Results

After detailed baseline assessment, 48 adolescents were allocated to the DCD group, while 37 to the TD group. Demographics and baseline characteristics are presented in Table 1. Independent samples *t*-tests revealed that pre intervention there were significant differences between the two groups in MABC-2, shuttle-run score, and PAQ-A. Four participants discontinued the intervention, five were lost post intervention, and four more were lost at 12 weeks follow-up (Supplementary Figure 1). In total, 76 participants (88%) completed the study. Details on study feasibility are available in Supplementary material.

**TABLE 1:** Characteristics of participants at each time point.

	TD			DCD		
	**Pre (*n* = 37)**	**Post (*n* = 36)**	**Follow (*n* = 32)**	**Pre (*n* = 37)**	**Post (*n* = 48)**	**Follow (*n* = 41)**
Age; M (SD)	13.9 (0.3)	14.1 (0.3)	13.3 (3.5)	13.9 (0.3)	14.1 (0.3)	13.7 (2.4)
Height/m; M (SD)	1.65 (0.1)	1.65 (0.1)	1.66 (0.1)	1.65 (0.1)	1.62 (0.1)	1.62 (0.1)
Weight/kg; M (SD)	58.5 (12.8)	58.8 (13.5)	61 (14.3)	58.5 (12.8)	61.2 (13.6)	61.6 (14.9)
BMI; M (SD)	21.4 (3.9)	21.5 (4.1)	22.1 (4.4)	21.4 (3.9)	23.4 (5.1)	23.3 (5.3)
PAQ-A						
M (SD)	2.3 (0.5)	2.2 (0.5)	2.3 (0.6)	2.3 (0.5)	2.2 (0.5)	2.2 (0.5)
Median (min–max)	2.2 (1.1–3.5)	2.2 (1.4–3.3)	2.2 (1.1–3.4)	2.2 (1.1–3.5)	2.1 (1.4–3.2)	2.2 (1.2–3)
**MABC-2**						
M(SD)	33.5 (15.1)	32.6 (14.4)	32 (14.6)	5.5 (3.2)	5.6 (3.3)	5.2 ± 3.3
Median (min–max)	25 (16–63)	25 (16–63)	25 (16–63)	5.0 (0.1–9.0)	5 (0.1–9)	5 (0.1–9)
**CHU9D**						
M (SD)	0.9 (0.1)	0.9 (0.1)	0.9 (0.1)	0.9 (0.1)	0.9 (0.1)	0.9 (0.1)
Median (min–max)	0.9 (0.7–1)	0.9 (0.6–1)	0.9 (0.7–1)	0.9 (0.7–1)	0.9 (0.7–1)	0.9 (0.7–1)
**HSPC**						
**Median (min–max)**						
Scholastic	17 (7–24)	17 (11–24)	17.5 (11–24)	17 (7–24)	16.5 (9–23)	16 (10–23)
Social	18 (10–24)	17 (8–24)	18 (12–24)	18 (10–24)	18 (8–22)	17 (11–22)
Athletic	14 (7–23)	15 (8–24)	15 (6–24)	14 (7–23)	13 (6–20)	13 (6–19)
Physical appearance	15 (7–24)	15 (8–24)	15 (6–24)	15 (7–24)	13 (6–20)	13 (6–19)
Behavioral conduct	14 (6–24)	15.5 (6–25)	16.5 (11–24)	14 (6–24)	16 (6–24)	16 (6–24)
Global self-worth	18 (11–24)	18 (12–24)	18 (12–24)	18 (11–24)	18 (11–24)	18 (7–24)

TD, typically developed; DCD, developmental coordination disorder; BMI, body mass index; PAQ-A, physical activity audit for adolescents; MABC, movement assessment battery for children-2nd edition; CHU9D, child health utility 9D; HSPC, Harter’s self-perception profile for children.

### Motor and cognitive performance

**FIGURE 2:**
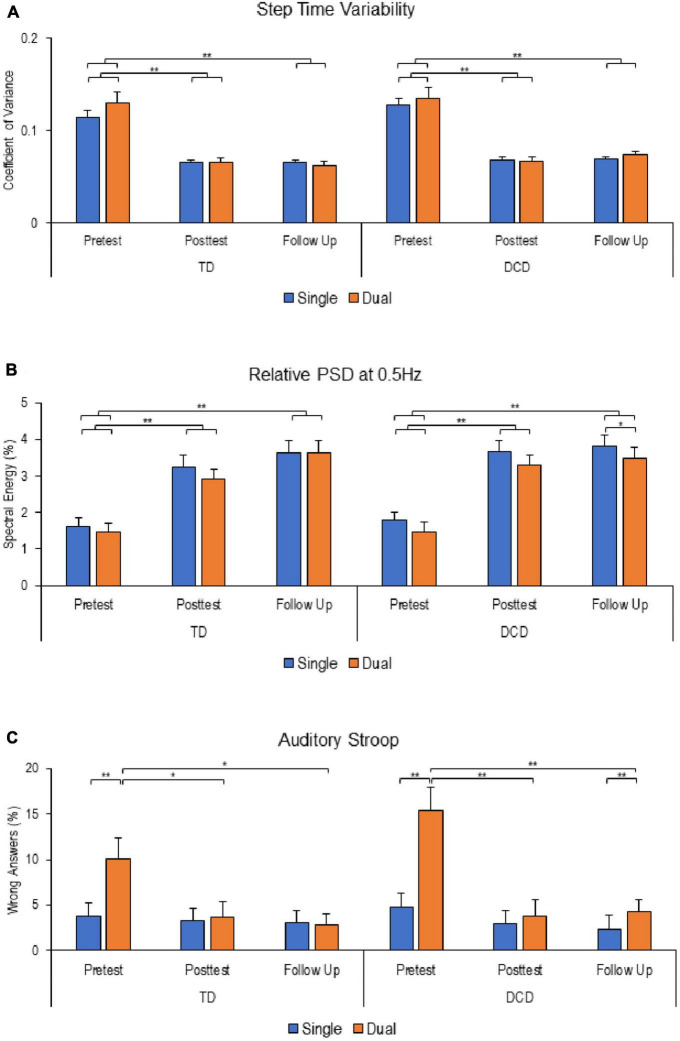
Stepping and auditory Stroop performance at different time points. **(A)** Step time viability. **(B)** Relative powers spectral density at 0.5 Hz. **(C)** Percentage of wrong answers in the Auditory Stroop task. Except for relative power spectral density (PSD), reduced values reflect improved performance. Error bars represent standard error of the mean (*P < 0.05; **P < 0.01).

The results of performance in the stepping motor and auditory Stroop tasks are illustrated in Figure 2. For step time variability, mixed-design ANOVA revealed a significant main effect of Time [F (2, 82) = 88.222; *P* < 0.001; η_*p*_^2^ = 0.683]. There were no significant main effects of Task [F (1,41) = 1.747, *P* = 0.194; η_*p*_^2^ = 0.041], Group [F (1, 41) = 1.713, *P* = 0.198; η_*p*_^2^ = 0.04], or interactions. In other words, step time variability was significantly higher at baseline assessment compared to the post-intervention and follow-up assessments for both groups and tasks (Figure 2A).

For stepping frequency adherence, measured by relative PSD at 0.5 Hz, there were significant main effects of Task [F (1, 43) = 11.486; *P* = 0.002; η_*p*_^2^ = 0.211] and Time [F (2, 86) = 57.641; *P* < 0.001; η_*p*_^2^ = 0.573], with no significant main effect of Group [F (1, 43) = 0.318, *P* = 0.576; η_*p*_^2^ = 0.007] or interactions. Relative PSD at 0.5 Hz was significantly lower at baseline reflecting more variability in performance compared to post-intervention and follow-up for both groups (Figure 2B).

Analysis of participants performance in the auditory Stroop task revealed significant main effects of Time [F (2, 124) = 9.868, *P* < 0.001; η_*p*_^2^ = 0.137] and Task [F (1, 62) = 36.953, *P* < 0.001; η_*p*_^2^ = 0.373], with significant interactions between Time*Task [F (2, 124) = 27.573, *P* < 0.001; η_*p*_^2^ = 0.308] and Task*Group [F (1, 62) = 4.436, *P* = 0.039; η_*p*_^2^ = 0.067]. Further analysis revealed that both groups made more errors under dual-task conditions at baseline assessment. However, at follow-up assessment, a significant difference in performance between single- and dual-task was observed only in the DCD group only. Interestingly, both groups demonstrated improved performance in dual-tasking at post-intervention and follow-up compared to baseline assessment (Figure 2C).

### PFC activation—fNIRS

**FIGURE 3:**
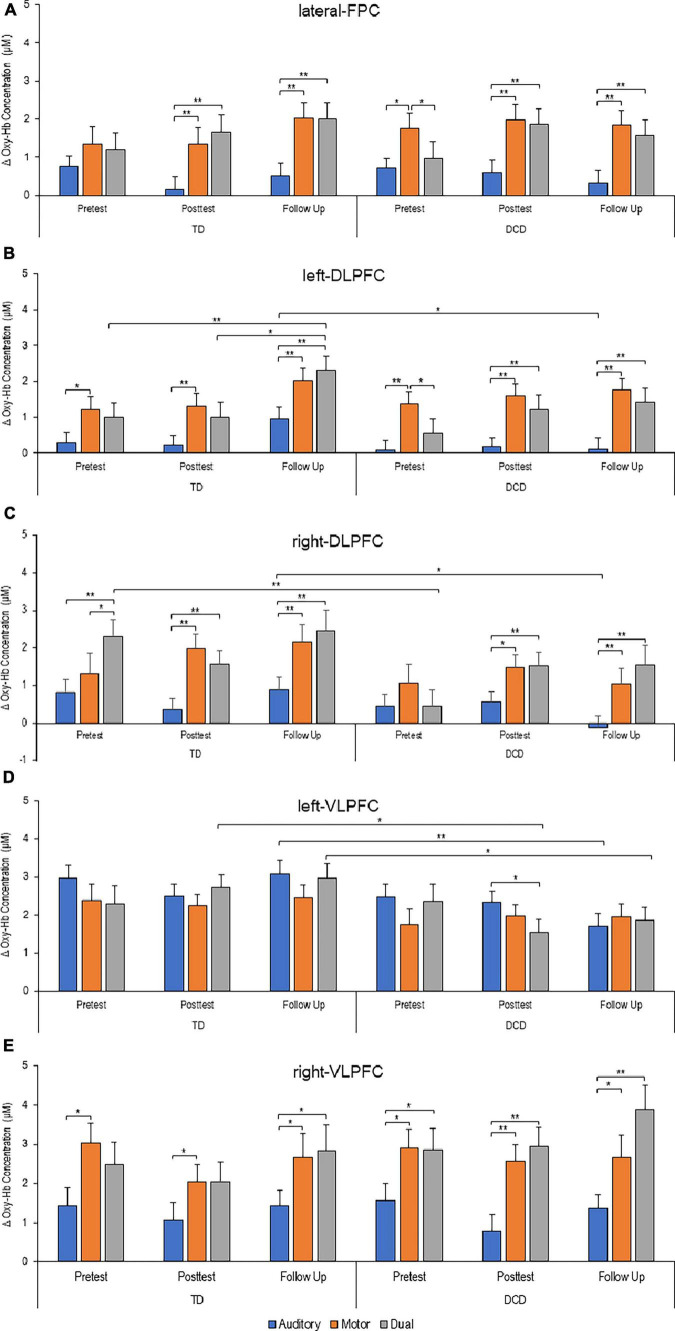
Task-related elative changes in oxygenated (Oxy) hemoglobin concentration in different areas within the prefrontal cortex (PFC) at different time points (Δ Oxy-Hb in μM). **(A)** Lateral frontopolar cortex. **(B)** Left dorsolateral prefrontal cortex. **(C)** Right dorsolateral prefrontal cortex. **(D)** Left ventrolateral prefrontal cortex. **(E)** Right ventrolateral prefrontal cortex. Increased values reflect increased cortical activation. Error bars represent standard error of the mean (*P < 0.05; **P < 0.01).

Functional near-infrared spectroscopy revealed significant changes in relative Oxy-Hb concentrations related to Task, Time, and Group in the overall PFC. Average activation comparisons across different regions within the PFC are illustrated in Figure 3.

In the lateral FPC, mixed-design ANOVA revealed a significant main effect of Task [F (2, 130) = 36.642; *P* < 0.001; η_*p*_^2^ = 0.683] as well as a significant Task*Time interaction [F (4, 265) = 3.134; *P* = 0.015; η_*p*_^2^ = 0.047]. In both groups, when comparing different tasks, pairwise comparisons indicated that post-intervention and at follow-up assessments, the lateral FPC exhibited reduced activation during the auditory Stroop task compared to stepping and dual-tasking. However, at baseline assessment, in the DCD group specifically, the lateral FPC showed higher activation in the stepping task compared to the auditory Stroop task and dual-tasking (Figure 3A).

In the left DLPFC, mixed-design ANOVA revealed significant main effects of Task [F (2, 128) = 49.905; *P* < 0.001; η_*p*_^2^ = 0.438] and Time [F (2, 128) = 3.219; *P* = 0.043; η_*p*_^2^ = 0.048] with no significant differences between groups or interactions. Pairwise comparisons showed that the left DLPFC exhibited increased activation during stepping compared to auditory Stroop for both groups and at each time point (Figure 3B). Additionally, during dual-tasking, the left DLPFC exhibited increased activation compared to the auditory Stroop task at post-intervention and follow-up assessments in the DCD group, and only at the follow-up assessment in the TD group. However, at baseline, the left DLPFC exhibited greater activation during stepping compared to dual-tasking, specifically in the DCD group.

Regarding the intervention effect, it was only significant during dual-tasking and only for the TD group. In this group, the left DLPFC demonstrated increased activation at the post-intervention and follow-up assessments compared to the baseline assessment.

Finally, the only significant difference between the two groups in terms of left DLPFC activation was observed at the follow-up assessment while participants performed the auditory Stroop task (Figure 3B).

In the right DLPFC, mixed-design ANOVA revealed a significant main effect of Task [F (2, 128) = 39.628; *P* < 0.001; η_*p*_^2^ = 0.386] and a significant Task*Time*Group interaction [F (4, 252) = 2.782; *P* = 0.027; η_*p*_^2^ = 0.036] with no significant differences between groups or interactions. Pairwise comparisons showed that the right DLPFC exhibited reduced activation during auditory Stroop performance compared to stepping and dual-tasking at all three time points for the TD group. For the DCD group, the right DLPFC showed reduced activation during auditory Stroop performance compared to stepping and dual-tasking, but only at the post-intervention and follow-up assessments (Figure 3C).

Significant between-group differences in right DLPFC activation were observed during dual-tasking at baseline, with the TD group showing higher activation compared to the DCD group. Similarly, during the auditory Stroop task at follow-up, the TD group exhibited greater right DLPFC activation compared to the DCD group.

In the left VLPFC, mixed-design ANOVA revealed significant main effects of Task [F (2, 130) = 3.325; *P* = 0.039; η_*p*_^2^ = 0.049] and Group [F (1, 65) = 5.968; *P* = 0.017; η_*p*_^2^ = 0.084] as well as a significant Task*Time*Group interaction [F (4, 260) = 2.735; *P* = 0.029; η_*p*_^2^ = 0.012]. Pairwise comparisons revealed significant increase in left VLPFC activation during auditory Stroop, but only for the DCD group and specifically at the post-intervention assessment (Figure 3D).

Significant between-group differences in left VLPFC activation were observed during dual-tasking at the post-intervention and follow-up assessments, with the TD group showing higher activation compared to the DCD group. Additionally, during the auditory Stroop task at follow-up, the TD group exhibited greater left VLPFC activation compared to the DCD group.

In the right VLPFC, mixed-design ANOVA revealed a significant main effect of Task [F (2, 118) = 41.575; *P* < 0.001; η_*p*_^2^ = 0.413]. Pairwise comparisons showed that, in the TD group, the right VLPFC demonstrated lower activation during auditory Stroop performance compared to stepping at all three time points. Additionally, the difference in activation between auditory Stroop and dual-tasking was only significant at the follow-up assessment. In contrast, for the DCD group, the right VLPFC showed increased activation during both stepping and dual-tasking compared to auditory Stroop at all three time points (Figure 3E).

### Whole brain imaging—MRI

A total of 35 DCD (73%) and 24 TD (65%) who were eligible consented to MRI assessment, 7 were not assessed at week 7 (1 TD, 6 DCD) due to incidental findings (*n* = 2), claustrophobia (*n* = 1) and loss post intervention [non-completion (*n* = 3) and extended delay in post-intervention assessment (*n* = 1)].

Across both groups, significant positive activation was observed during stepping compared to rest (FWE-correlation; *P* < 0.05) in the central sensory-motor cortex and in superior part of the cerebellum (Supplementary Figure 2). However, no significant effect of Group (TD vs. DCD), Time (baseline vs. post-intervention), or their interaction was found in relation to this activation pattern.

**FIGURE 4:**
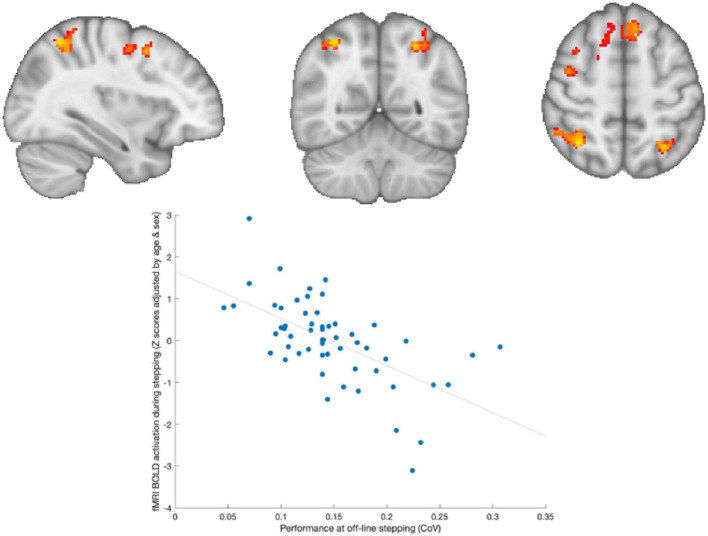
Brain activation associated with out-of-scanner stepping. Adolescents who showed greater activation in frontoparietal areas during stepping were those with lower variability in out-of-scanner stepping performance.

During the baseline assessment, a significant negative correlation was observed across both groups between brain activation during in-scanner stepping and performance during out-of-scanner stepping (as depicted in Figure 4). This means that adolescents who exhibited greater positive activity in frontoparietal regions of the brain had lower variability in their performance during out-of-scanner stepping tasks.

**FIGURE 5:**
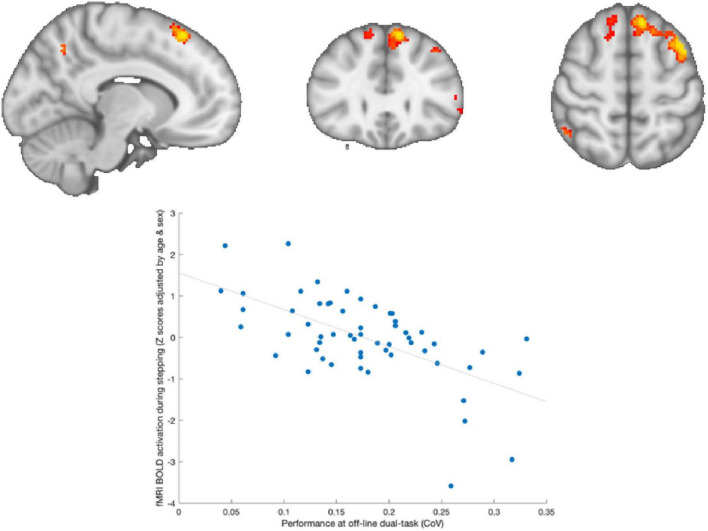
Brain activation associated with out-of-scanner dual-tasking. Adolescents who showed greater activation in frontal areas (left superior- and medial-frontal cortex) during stepping were those with lower variability in out-of-scanner dual-task performance.

Similarly, we observed a significant negative correlation across both groups between brain activation during in-scanner stepping and performance during out-of-scanner dual-task performance (as shown in Figure 5). This indicates that adolescents who exhibited greater positive activity in frontal regions of the brain had less variability in their performance during out-of-scanner dual-tasking.

At the 7 weeks post-intervention assessment, we did not find any statistically significant effects of training on brain structure and function, nor did we observe a significant interaction between Time and Group.

When examining brain structural measures, we did not find any significant effects of Time or Group. However, we did find baseline correlations between diffusion MRI metrics and individual differences in dual-task performance (Supplementary Figure 3).

## Discussion

In this study, we present the findings of a 7 weeks exercise intervention program aimed at improving motor skills in young people with DCD. This trial is notable for its relatively large sample size and the integration of brain imaging and motion capture techniques to investigate the acquisition of motor skills and the underlying mechanisms in adolescents who fell within the lowest quartile of fitness parameters. The inclusion of participants both with and without DCD adds to the study’s significance, allowing for a comprehensive examination of motor skill acquisition across a range of individuals.

Our results indicate that adolescents with DCD performed the novel stepping task similarly to their peers who had lower levels of fitness. This suggests that individuals with DCD have the capacity to learn and improve their motor performance. However, distinct differences in cortical activation patterns within the PFC were observed between the two groups at different time points and task conditions. Furthermore, we identified a relationship between MRI brain structure and function measures and initial performance in the novel stepping task, both in single-task and dual-task conditions.

The findings of this study offer not only valuable insights into the process of acquiring motor skills in adolescents but also have implications for the development of effective interventions and strategies aimed at improving motor skills and enhancing motor learning in individuals with DCD. In the subsequent sections, we will delve into these insights and discuss their implications in greater detail.

### Behavioral outcomes

In dual-task paradigms, it is common to compare the performance of a balance or walking task when performed alone versus when combined with a concurrent cognitive task (Schott, 2019). In this study, we compared the performance of a novel rhythmic stepping task performed alone to the same task performed concurrently with an auditory Stroop task. The participants were divided into two groups: adolescents with and without motor performance characteristics of DCD, who had similar fitness levels. Prior to training, both groups faced greater challenges in achieving and maintaining the stepping frequency when performing the task with the additional auditory Stroop, and they made more errors in the auditory task during dual-task conditions.

Following the training intervention, both groups exhibited improvements in performance in both the stepping and auditory Stroop tasks, as well as a reduction in dual-task interference in the auditory Stroop. These improvements were maintained even 7 weeks after the training had ended.

Interestingly, significant differences between single- and dual-task performance emerged at the follow-up assessment for the DCD group, but not at the posttest. At follow-up assessment, the DCD group encountered challenges in maintain stepping frequency close to the desired target and made more errors in the dual-task condition of the auditory Stroop task. These findings suggest that individuals with DCD rely on cognitive resources when performing a novel stepping task, indicating potential difficulties in motor learning. This later observation aligns with the view that DCD involves deficits in motor learning (Schoemaker and Smits-Engelsman, 2015), and adolescents with DCD may adopt different learning strategies to manage concurrent tasks due to difficulties in automatization (Tsai et al., 2009) or deficits in internal modeling (Wilson et al., 2017). Additionally, they tend to exhibit slower and more variable task performance compared to their peers without DCD (Mackenzie et al., 2008; Jelsma et al., 2021). Taken together, our findings support the notion that individuals with DCD experience persistence difficulties in feedforward and feedback control mechanisms, which impede motor learning and movement automatization processes.

### Brain imaging

Our study employed both fMRI and fNIRS to investigate the functional and structural neural correlates associated with individual differences in performance during stepping and dual tasking. While the fMRI results did not reveal significant differences in brain activation patterns within or between groups, the fNIRS measurements provided novel insights into the neural underpinnings of motor performance in response to training.

The baseline findings reveal that individuals with DCD exhibited increased activation in specific areas within the PFC, including the lateral-FPC, left-DLPFC, and right-VLPFC, during the stepping task compared to the auditory Stroop and dual-task conditions. However, in the dual-task condition, the DCD group showed increased activation only in the right-VLPFC compared to the auditory Stroop task. This increased activation suggests reduced automatization (Boyne et al., 2018) and a greater reliance on higher cognitive centers for motor control in individuals with DCD (Damasio et al., 1996; da Silva et al., 2017). It indicates that adolescents with DCD require more cognitive control to perform the stepping task.

In the TD group, increased activations were observed in the left-DLPFC and right-VLPFC during stepping compared to the auditory Stroop task, suggesting that TD adolescents with low level of fitness may also rely on cognitive resources to execute a novel stepping task. Additionally, the right-DLPFC exhibited increased activation under the dual-task condition compared to both stepping and the DCD group. However, in the dual-task condition, the absence of additional over-activation in the DCD group suggests a less efficient reconfiguration of neural networks compared to the TD group (Leone et al., 2017; Fuelscher et al., 2018).

After the intervention, the DCD group demonstrated increased activation in all areas within the PFC during both the stepping and dual-task conditions compared to the auditory Stroop task. The only exception was the left-VLPFC, where auditory Stroop led to increased activation compared to the dual-task condition. In the TD group, increased activation was observed in all PFC areas, except for the left-VLPFC, during stepping compared to the auditory Stroop task. Additionally, the dual-task condition exhibited increased activation compared to the auditory Stroop task in the lateral-FPC and right-DLPFC. Notably, the left-VLPFC showed higher activation in the dual-task condition for TD individuals compared to the DCD group. However, overall activation patterns in the PFC were similar between the two groups, suggesting that adolescents with DCD exhibited training-induced adaptations in brain function consistent with typical performance during a gross motor task.

At the follow-up assessment, both the DCD and TD groups displayed increased activation in all areas within the PFC during both the stepping and dual-task conditions compared to the auditory Stroop task. The only exception was again the left-VLPFC, where between-group difference emerged with the TD group demonstrating higher activation than the DCD group during auditory Stroop and dual-task conditions. This is notable as observed alongside the deficits in dual-task performance in the DCD group. Interestingly, the TD group also exhibited increased activation in the DLPFC bilaterally during the auditory Stroop compared to the DCD group. This suggests that the interference observed in the Stroop task for individuals with DCD might be related to a failure to suppress automatically processed stimuli (Christensen et al., 2011). Furthermore, the TD group showed increased activation in the left-DLPFC during the dual-task condition at follow-up compared to baseline, indicating more efficient utilization of neural networks compared to the DCD group (Leone et al., 2017; Fuelscher et al., 2018).

These findings indicate that adolescents with low motor competence, such as those with DCD, exhibit distinct prefrontal activation responses during the learning and performance of a motor task, particularly when the task complexity is increased by additional concurrent cognitive tasks. This pattern of PFC activation may contribute to their tendency to avoid participating in physical activities and sports, as well as their reported lower enjoyment and greater fatigue when engaging in such activities, as previous research has suggested (Cairney et al., 2010).

The present study also revealed intriguing findings. However most interesting is the follow-up period where greater activity in the prefrontal areas was observed alongside better maintained performance, suggesting that the DCD group lose the ability to generate brain activity compared to their peers when not practicing motor tasks. Regarding the relationship between brain activation during in-scanner stepping and subsequent motor performance during out-of-scanner tasks, both in the single- and dual-task conditions. Specifically, we found that adolescents who exhibited greater activations within frontoparietal cortical networks had lower variability in their performance during out-of-scanner stepping. The frontoparietal regions are known to be involved in motor planning, execution, and sensorimotor integration processes (Marek and Dosenbach, 2018). The observed negative correlation suggests that individuals with higher activation in these regions have more efficient motor control mechanisms, resulting in better motor performance during subsequent out-of-scanner tasks.

Furthermore, we found that adolescents who exhibited greater positive activity in frontal regions had less variability in their performance during out-of-scanner dual-tasking. The frontal regions, particularly the prefrontal cortex, are associated with cognitive control, attentional processes, and task coordination (Fan et al., 2005; Leone et al., 2017). The negative correlation suggests that individuals with higher activation in frontal regions during in-scanner stepping may possess better cognitive control abilities, allowing them to effectively manage the additional cognitive demands imposed by the dual-task condition. This may lead to improved dual-task performance with reduced interference between motor and cognitive components.

The findings from brain MRI measures in this study showed no significant differences between the DCD and TD groups, but, rather a continuum as previously described in the relationship of higher activity relating to higher motor performances. This contrasts with some previous studies that have reported different patterns of brain activity between individuals with DCD and typically developing individuals at rest (McLeod et al., 2014) or during various motor and cognitive tasks involving the upper limb (Querne et al., 2008; Zwicker et al., 2011).

Additionally, the present study did not find evidence of functional or structural brain changes over time in relation to the intervention. These results suggest that there may be a continuum of individuals with motor difficulties (Gomez and Sirigu, 2015) rather than distinct cutoffs when considering functional brain imaging specifically during stepping tasks.

It is important to note that the absence of significant group differences in brain MRI measures does not necessarily imply that there are no underlying neural differences between individuals with DCD and typically developing individuals. It is possible that the similarly low level of fitness between or that the specific motor task used in the study did not elicit robust differences in brain activation between the groups. Further research including a wider range of adolescents and utilizing a variety of motor tasks is warranted to better understand the neural mechanisms underlying DCD and to explore potential subgroups or continuums within individuals with motor difficulties.

### General discussion

The fNIRS results indicated that, prior to the intervention, there were differences in PFC activation patterns between the TD group and the DCD group specifically during tasks with a physical component. This highlights DCD as primarily a motor dysfunction. However, it is important to note that the stepping task used in the study involved visual processing, as participants had to attend to a screen and follow directional instructions.

Seven weeks after the intervention, a group difference in PFC activation was observed during the auditory Stroop task, suggesting that the intervention elicited motor learning consistent with TD brain function in individuals with DCD during the performance of the specifically trained physical task. The effects of the learning were mainly seen in motor task performance and did not transfer to the non-trained auditory task or dual-tasking.

During the auditory Stroop task, both groups showed increased activation compared to the start of the task across all sessions, indicating that all participants engaged with the task and recruited neural resources to accomplish it. Group differences in the cognitive task were negligible before the intervention. However, group differences in the performance of the auditory Stroop task were negligible before as well as after the intervention.

Compared to the DCD group, individuals in the TD group showed less dual-task interference after training and exhibited more activation within the PFC while performing the auditory Stroop task. This suggests that the TD group benefited more from the training, resulting in increased efficiency in utilizing neural networks involving the PFC in general.

The motor findings suggest a reduced attentional focus in the DCD group, consistent with previous studies (Damasio et al., 1996; Damasio and Carvalho, 2013). It is proposed that homeostatic disturbances caused by activity create a conflict in resource allocation between interoceptive responses and cognitive states, both of which rely on prefrontal regulatory functioning (da Silva et al., 2017). The altered PFC activation pattern observed in individuals with DCD may reflect reduced cognitive resources affecting interoceptive input and cognitive factors during task learning and performance (da Silva et al., 2017). Perceptual factors are known to contribute to decisions about when to terminate exercise and are strong predictors of physical activity levels in both young people and adults (Ells et al., 2018). Therefore, these observations are important and warrant further investigation, particularly in the context of lower activity levels and reduced enjoyment of physical activity and exercise observed in young people with DCD.

## Conclusion

Taken together, our findings suggest that adolescents with DCD and low motor competence may have limited transfer of brain activity to tasks that are not specifically trained, and maintaining task performance when a task is not trained over time. This suggests that their ability to generalize motor skills to different contexts or tasks and maintain skills may be compromised. To address these challenges, strategies can be implemented to reduce the complexity of tasks or modify the environment in which physical activities and sports take place, especially when learning new motor skills, and strategies to top up tasks with training bursts over time.

Furthermore, our findings also highlight the importance of frontoparietal regions in facilitating motor control and cognitive processes necessary for motor performance in adolescents with low level of fitness. Understanding these neural correlates of motor performance can have implications for designing targeted interventions and strategies to improve motor skills in individuals with motor disorders, such as DCD. By identifying specific brain regions associated with successful motor performance, interventions can be tailored to enhance the functioning of these regions and promote better motor outcomes.

Consequently, interventions that aim to enhance brain activity through a range of tasks may offer opportunities to increase participation in physical activity and sports among adolescents with low motor competence. These interventions could involve incorporating cognitive exercises or training programs that target the PFC cortex and other relevant brain regions involved in motor control and learning.

## Data availability statement

The raw data supporting the conclusions of this article will be made available by the authors, without undue reservation.

## Ethics statement

The study was reviewed and approved by local Institutional Review Board (UREC 161033). Written informed consent to participate in this study was provided by the participants’ legal guardian/next of kin.

## Author contributions

EA-Y was involved in project administration, data analysis, drafted the manuscript, and edited and reviewed the manuscript. PE was involved in project conceptualization, funding acquisition, planned and performed experiments, project administration, and editing the manuscript. BW was involved in project administration, collected data, and planned and performed experiments. SJ was involved in project administration, collected and analyzed data, drafted the manuscript, edited and reviewed the manuscript, and planned and performed experiments. Y-CL and DS performed experiments and collected data. PS and MI was involved in data analysis. AM was involved in project administration and performed experiments. JC was involved in project conceptualization, funding acquisition, and project administration. AD, SK, and HI were involved in project conceptualization and funding acquisition. TW was involved in project conceptualization, data analysis, and funding acquisition. HJ-B and HD were involved in project conceptualization, project administration, data analysis, funding acquisition, and supervision reviewing and editing the manuscript. HA was involved in project conceptualization, project administration, data analysis, and funding acquisition. All authors contributed to the article and approved the submitted version.
