# Corrigendum: Motor learning in developmental coordination disorder: behavioral and neuroimaging study

**DOI:** 10.3389/fnins.2024.1433125

**Published:** 2024-07-17

**Authors:** Emad Al-Yahya, Patrick Esser, Benjamin D. Weedon, Shawn Joshi, Yan-Ci Liu, Daniella N. Springett, Piergiorgio Salvan, Andy Meaney, Johnny Collett, Mario Inacio, Anne Delextrat, Steve Kemp, Tomas Ward, Hooshang Izadi, Heidi Johansen-Berg, Hasan Ayaz, Helen Dawes

**Affiliations:** ^1^School of Health Sciences, University of Nottingham, Nottingham, United Kingdom; ^2^School of Rehabilitation Sciences, University of Jordan, Amman, Jordan; ^3^Centre for Movement, Occupation and Rehabilitation Services, Oxford Brookes University, Oxford, United Kingdom; ^4^School of Biomedical Engineering, Science and Health Systems, Drexel University, Philadelphia, PA, United States; ^5^College of Medicine, Drexel University, Philadelphia, PA, United States; ^6^School and Graduate Institute of Physical Therapy, College of Medicine, National Taiwan University, Taipei City, Taiwan; ^7^Department for Health, University of Bath, Bath, United Kingdom; ^8^Wellcome Centre for Integrative Neuroimaging, Nuffield Department of Clinical Neurosciences, University of Oxford, Oxford, United Kingdom; ^9^Research Center in Sports Sciences, Health Sciences and Human Development, University of Maia, Porto, Portugal; ^10^Insight SFI Research Centre for Data Analytics, Dublin City University, Dublin, Ireland; ^11^School of Engineering, Computing and Mathematics, Oxford Brookes University, Oxford, United Kingdom; ^12^Department of Psychological and Brain Sciences, College of Arts and Sciences, Drexel University, Philadelphia, PA, United States; ^13^Drexel Solutions Institute, Drexel University, Philadelphia, PA, United States; ^14^Department of Family and Community Health, University of Pennsylvania, Philadelphia, PA, United States; ^15^Center for Injury Research and Prevention, Children's Hospital of Philadelphia, Philadelphia, PA, United States; ^16^NIHR Exeter BRC, Medical School, University of Exeter, Exeter, United Kingdom

**Keywords:** developmental coordination disorder, motor control, prefrontal cortex, frontoparietal networks, fNIRS, MRI

In the published article, there was an error in Table 1. The number of DCD participants at baseline appeared as Pre (*n* = 37) and at Follow as (*n* = 36). The correct numbers should be Pre (*n* = 48) and Follow (*n* = 41). The values of MABC-2 scores for the DCD group appeared as M (SD) = 33.5 (15.1) the correct value should be M (SD) = 5.5 (3.2); The Median (min–max) of MABC-2 scores for the DCD group appeared as 25 (16–63) while it should be 5.0 (0.1–9). The corrected Table 1 and its caption “Characteristics of participants at each time point” appear below.

**Table 1:** Characteristics of participants at each time point.

	**TD**			**DCD**		
	**Pre (*****n*** = **37)**	**Post (*****n*** = **36)**	**Follow (*****n*** = **32)**	**Pre (*****n*** = **48)**	**Post (*****n*** = **44)**	**Follow (*****n*** = **41)**
Age; M (SD)	13.9 (0.3)	14.1 (0.3)	13.3 (3.5)	13.9 (0.3)	14.1 (0.3)	13.7 (2.4)
Height/m; M (SD)	1.65 (0.1)	1.65 (0.1)	1.66 (0.1)	1.65 (0.1)	1.62 (0.1)	1.62 (0.1)
Weight/kg; M (SD)	58.5 (12.8)	58.8 (13.5)	61 (14.3)	58.5 (12.8)	61.2 (13.6)	61.6 (14.9)
BMI; M (SD)	21.4 (3.9)	21.5 (4.1)	22.1 (4.4)	21.4 (3.9)	23.4 (5.1)	23.3 (5.3)
**PAQ-A**						
M (SD)	2.3 (0.5)	2.2 (0.5)	2.3 (0.6)	2.3 (0.5)	2.2 (0.5)	2.2 (0.5)
Median (min–max)	2.2 (1.1–3.5)	2.2 (1.4–3.3)	2.2(1.1–3.4)	2.2 (1.1–3.5)	2.1(1.4–3.2)	2.2 (1.2–3)
**MABC-2**						
M (SD)	33.5 (15.1)	32.6 (14.4)	32 (14.6)	5.5 (3.2)	5.6 (3.3)	5.2 ± 3.3
Median (min–max)	25 (16–63)	25 (16–63)	25 (16–63)	5.0 (0.1-9.0)	5 (0.1–9)	5 (0.1–9)
**CHU9D**						
M (SD)	0.9 (0.1)	0.9 (0.1)	0.9 (0.1)	0.9 (0.1)	0.9 (0.1)	0.9 (0.1)
Median (min–max)	0.9 (0.7–1)	0.9 (0.6–1)	0.9 (0.7–1)	0.9 (0.7–1)	0.9 (0.7–1)	0.9 (0.7–1)
**HSPC**						
**Median (min–max)**						
Scholastic	17 (7–24)	17 (11–24)	17.5 (11–24)	17 (7–24)	16.5 (9–23)	16 (10–23)
Social	18 (10–24)	17 (8–24)	18 (12–24)	18 (10–24)	18 (8–22)	17 (11–22)
Athletic	14 (7–23)	15 (8–24)	15 (6–24)	14 (7–23)	13 (6–20)	13 (6–19)
Physical appearance	15 (7–24)	15 (8–24)	15 (6–24)	15 (7–24)	13 (6–20)	13 (6–19)
Behavioral conduct	14 (6–24)	15.5 (6–25)	16.5 (11–24)	14 (6–24)	16 (6–24)	16 (6–24)
Global self-worth	18 (11–24)	18 (12–24)	18 (12–24)	18 (11–24)	18 (11–24)	18 (7–24)

TD, typically developed; DCD, developmental coordination disorder; BMI, body mass index; PAQ-A, physical activity audit for adolescents; MABC, movement assessment battery for children-2nd edition; CHU9D, child health utility 9D; HSPC, Harter's self-perception profile for children.

The authors apologize for this error and state that this does not change the scientific conclusions of the article in any way. The original article has been updated.

