# Supplementary material for: Motor learning in developmental coordination disorder: behavioral and neuroimaging study
Source: Front Neurosci. 2023 Jun 22;17:1187790. doi: 10.3389/fnins.2023.1187790 (PMC10323432; doi:10.3389/fnins.2023.1187790)
Supplement: Supplementary file 1 [file Data_Sheet_1.docx]

# Supplementary Material

## Feasibility and Participants Characteristics

Between February 2017 and March 2018, 293 (25%) potentially eligible individuals were identified from an existing Oxfordshire school cohort (n= 1174) and 86 (29%) recruited. This was 43% greater than the planned sample size, enabling greater statistical power within study budget(Esser et al. 2019). Group allocation resulted in 48 assigned to DCD and 37 to TDC groups. Figure S1. shows participant flow. Four participants discontinued the intervention, 5 were lost to post intervention (week 7) and an additional 4 participants were lost at 12-week follow-up. In total, 76 participants (88%) completed the study. There were 4 adverse events, none deemed trial-related; injury to arm, diagnosis of Bell’s Palsy, pre-indicator of Anorexia, and referral for later diagnosed exercise-induced asthma.

Feasibility success parameters were recruitment rate, intervention adherence (target >75%) primary outcome completion (target >80%), data quality (completion of outcomes) and resource needs (staff time, assessment time, space needs). Experience and satisfaction of participants and their parent/carer was assessed via an open questionnaire.

Of those who completed, primary outcome completion rate was 95.5% with other outcome measures reflecting the same completion rate (95%).

All participants rated trial experience positively, 38% rating it excellent, and most perceived improvement in confidence (54%) and engagement with sport (63%). Most parents reported an increase in their child’s confidence (68%) and all rated their child’s experience positively, with 43% rating it excellent. Participants suggested improvements to timing of intervention to enable them to have lunch with their friends and parents suggested that information post intervention should be available of sport opportunities for their child (type of sport, cost and timetables).


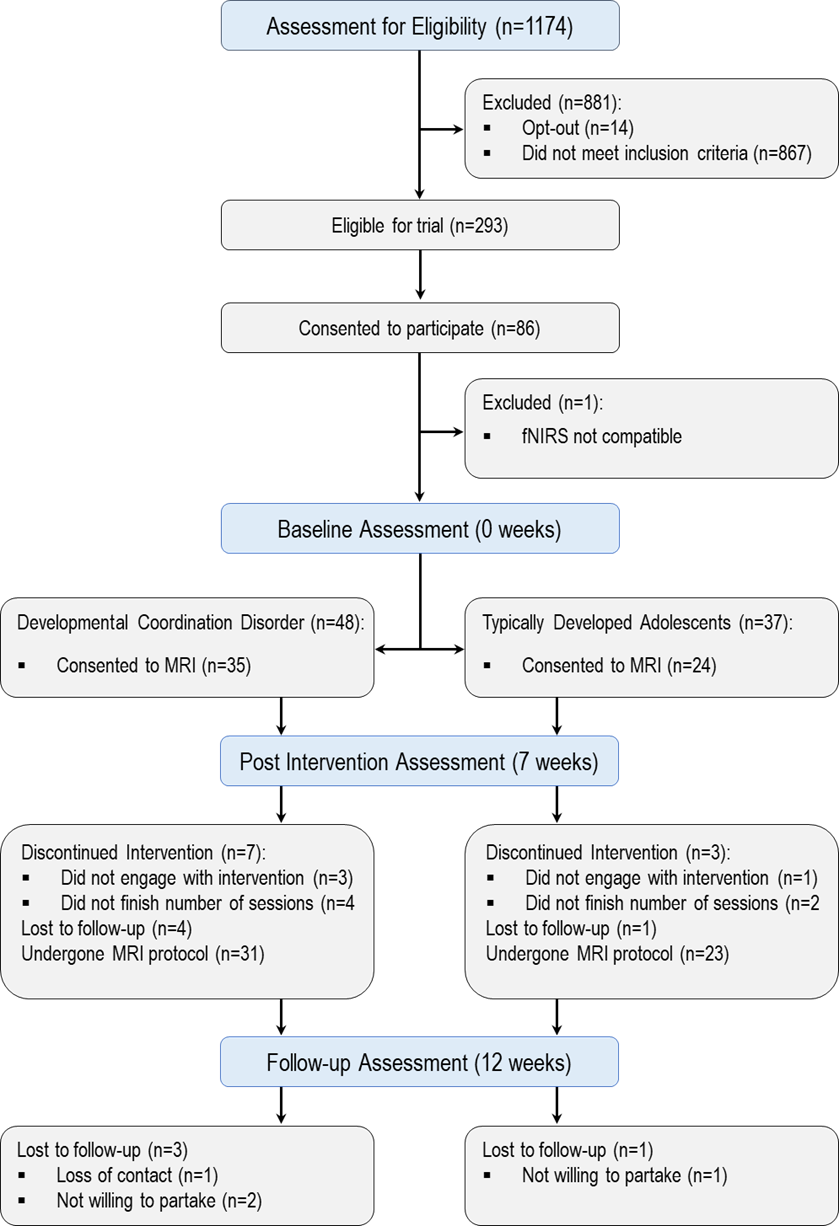


Figure S1. Participant flow diagram

The trial exceeded recruitment rate, the 80% target for completion of outcome measures (95%), the 75% target for adherence (88%). There were no safety concerns and trial experience was rated positively by participants and parents. These results support school based approaches that have been suggested to support individuals with DCD (Camden et al. 2019), and to have the potential to promote physical and psychological health in the long run. In line with previous findings (Kemp 2013), half of participants perceived improvement in their confidence, and nearly two thirds perceiving better engagement in sports following the study. Parents also reported a general increase in their child’s confidence.

## MRI

A total of 35 DCD (73%) and 24 TD (65%) who were eligible consented to MRI assessment, seven were not assessed at week 7 (1 TDC, 6 DCD) due to incidental findings (n=2), claustrophobia (n=1) and loss post intervention (non-completion (n=3) & extended delay in post-intervention assessment (n=1).

Due to head-motion and/or MRI artifact, a different number of participants with usable data was present depending on time point of interest and MRI modality (Supplemantary Table 1).

|  | T1w | task-fMRI | DW-MRI |
| --- | --- | --- | --- |
| Time point 1 | 58 | 57 | 55 |
| Time point 1&2 | 50 | 50 | 48 |

Supplementary Table 1: Number of participants with useable MRI data given modality.

### MRI acquisition

MRI-scans were carried out at WIN using a 3T Siemens Magnetom Prisma (Erlangen, Germany) scanner with a 32-channel head coil. The MR-protocol comprised both functional and structural sequences, takes approximately 30 minutes, and included:

1. Task-based fMRI scan, using a multi-band echo-planar imaging sequence (TR = 1355ms, TE = 32.40ms, FoV = 192mm, slice thickness = 2mm, 72 slices, voxel size 2x2x2mm^3^, acceleration factor = 4, ~6 min acquisition). During the task condition the participant was requested to tap their feet alternatingly at a fixed frequency of 0.5Hz for the duration of 30 seconds whereby a stimulus (LEFT/RIGHT) was visible for 1.5s followed by a 0.5s blank screen, programmed in Presentation (NBS, USA). The foot movement was measured via a bespoke MR-safe foot tapping device linked to potentiometers of which the positional angle is recorded in a bespoke LabVIEW programme (National Instruments, Ireland).
2. Anatomical T1-weighted structural scan, using a three-dimensional rapid gradient echo sequence (MPRAGE, TR = 1900ms, TE = 3.97ms, FoV = 192mm, FA = 8°, voxel size 1x1x1mm^3^, ~5.5min acquisition)
3. Diffusion-weighted images (DWI), using a multi-band echo planar imaging sequence (60 diffusion weighted directions, *b*-value = 1500s/mm^2^, four non-diffusion weighted images, *b*-value = 0s/mm^2^, TR = 2951ms, TE = 83.3ms, FoV = 204mm, voxel size 1.5x1.5x1.5mm^3^, acceleration factor = 4, ~3.30min acquisition). Another set of DWI data was collected with reverse-phase encoding direction (same parameters).

### MRI processing

MRI data was processed using FSL software (Jenkinson et al. 2012).

**Structural**

Pre-processing of T1w structural images was carried out using FSL ANAT (<https://fsl.fmrib.ox.ac.uk/fsl/fslwiki/fsl_anat>). Bias-field correction, brain extraction, tissue concentration estimation, and non-linear alignment to MNI152 standard-space T1 template were all carried out within this pipeline.

Grey matter (GM) tissue concentration was then “modulated” in order to carry out a voxel-based morphometry (VBM)-style analysis: GM maps were warped into MNI152 standard-space using the FSL FNIRT nonlinear warps (estimated within the FSL ANAT pipeline), multiplied by the Jacobian warp field, and spatially smoothed using a Gaussian kernel of sigma = 2mm (full-width at half maximum (FWHM) = 2 x 2.3 = 4.6mm).

**Task-functional MRI**

Task-fMRI data was processed using FSL FEAT (Woolrich et al. 2001). Functional volumes were corrected for geometric distortions due to susceptibility-induced field inhomogeneities and realigned to the first volume in the sequence using a six-parameter rigid body transformation to correct for motion. Structured artifacts were removed using independent component analysis (ICA) using FMRIB’s ICA-based X-noisefier (FIX) (Salimi-Khorshidi et al. 2014).

Task data was modelled according to the block design using a general linear model (GLM). The stepping blocks were modelled using a single explanatory variable (EV) that was then contrasted against baseline.

Images were spatially normalised by warping subject-specific images to the MNI152 standard-space T1 template. A Gaussian kernel of sigma = 2mm (FWHM = 2 x 2.3 = 4.6mm) was then applied at this stage in order to carry out the same level of spatial smoothing independent of head-size.

The resulting map of positive contrast of parameter estimates (COPE) was then fed to voxelwise statistical testing.

**Diffusion weighted MRI**

Diffusion Weighted (DW)-MRI data was first corrected for eddy currents, EPI distortions, inter- and intra-volume subject head motion, with outlier-slice replacement, using FSL Topup and Eddy (Andersson et al. 2003; Andersson and Sotiropoulos 2015). Diffusion Tensor Imaging (DTI) fitting was carried out with FSL DTIFIT (Behrens et al. 2007). FSL TBSS pipeline was then carried out (Smith et al. 2006). DTI maps of Fractional Anisotropy (FA) and Mean Diffusivity (MD) were aligned into a common space. Next, the mean FA image was created and thinned to create a mean FA skeleton which represents the centres of all tracts common to the group. Each subject's aligned FA data was then projected onto this skeleton. MD maps were also projected onto this skeleton using the transformation estimated from FA maps. The resulting FA and MD maps were fed to voxelwise statistical testing with Threshold-Free Cluster Enhancement (TFCE) with 2D optimization.

**Statistical analyses**

A finite set of research questions was tested across all the modalities and it is here summarised:

1. Is there a statistically significant difference between low-fit and DCD pupils (defined according to scores on the Movement Assessment Battery for Children (MABC)? (Tested as low-fit > DCD; and as DCD > low-fit).
2. Is there a significant longitudinal change? (Tested as a paired-difference in post-minus-pre changes being > 0).
3. Is there a significant group by time interaction? (Tested as group differences in longitudinal changes).
4. Is there a significant association with performance on the stepping task conducted outside the scanner? (Tested as a linear regression with performance (CoV) on the out-of-scanner stepping task).
5. Is there a significant association with performance during out-of-scanner dual-tasking? (Tested as a linear regression with performance (CoV) during out-of-scanner dual-tasking).
6. *Only* for task-fMRI: is there a significant association between brain activation during stepping and in-scanner performance? (Tested as a linear regression with performance (frequency and coefficient of variation (CoV)) during in-scanner stepping task).

Baseline cross-sectional analyses were carried out while adjusting for age and gender. Longitudinal analyses were carried out on subject paired-differences (post-minus-pre) while adjusting only for gender.

Voxelwise statistical inference was carried out through FSL randomise using a GLM with non-parametric permutation testing (Winkler et al. 2014). The level of statistical significance was assessed after 10,000 permutations and family-wise-error (FWE)-correction. TFCE with 2D optimization was carried out for TBSS analyses of DTI data; 3D TFCE was carried out otherwise. All FWE-corrected *P* values < 0.05 were considered significant. Although this process corrects for multiple comparisons across voxels, no correction across research questions or MRI modalities was applied. Where a statistically significant result was found, a subject-average value was extracted from the significant cluster and plotted in order to visualise the underlying data using scatter and/or box plots.


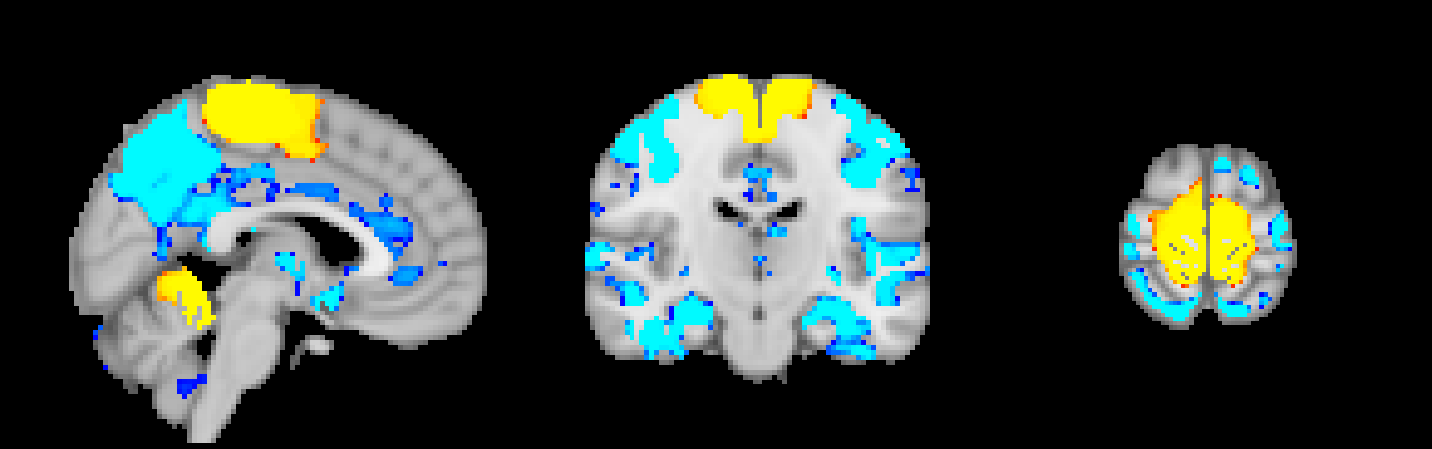


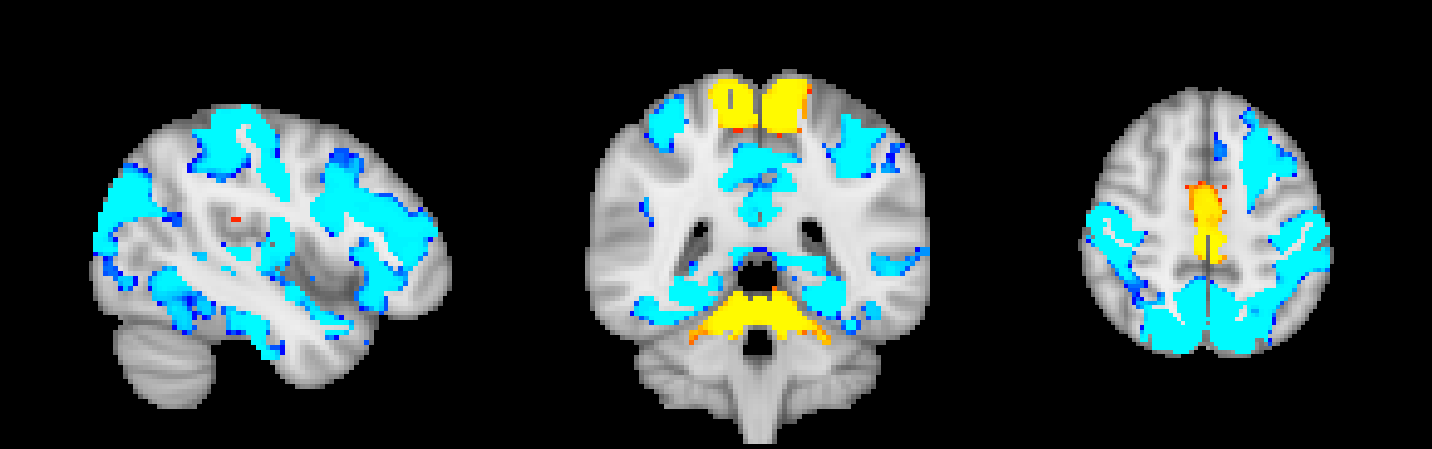


Figure S2. Group-level task-fMRI activation map for stepping task (Stepping vs. rest). Positive activation during stepping (stepping>rest) was found bilaterally in the central sensory-motor cortex and in the superior part of the cerebellum. Negative stepping activation (rest>stepping) was found in the default mode regions (anterior and posterior cingulate cortex), bilaterally in fronto-parietal areas, and in the parahippocampal cortices

**Diffusion weighted MRI**


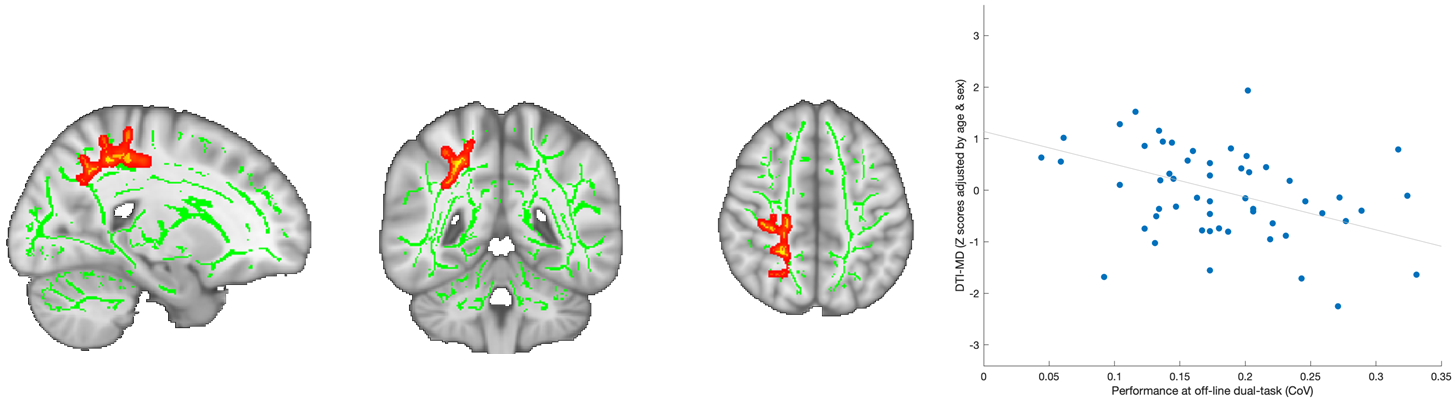


Figure S3. Inter-individual differences in white-matter microstructure were associated with performance during out-of-scanner dual-tasking. Greater DTI MD values negatively correlated with performance (CoV) during out-of-scanner dual-tasking

## References

Andersson JL, Sotiropoulos SN (2015) Non-parametric representation and prediction of single- and multi-shell diffusion-weighted MRI data using Gaussian processes. Neuroimage 122:166-176 doi: 10.1016/j.neuroimage.2015.07.067

Andersson JLR, Skare S, Ashburner J (2003) How to correct susceptibility distortions in spin-echo echo-planar images: application to diffusion tensor imaging. NeuroImage 20:870-888 doi: <https://doi.org/10.1016/S1053-8119(03)00336-7>

Behrens TEJ, Berg HJ, Jbabdi S, Rushworth MFS, Woolrich MW (2007) Probabilistic diffusion tractography with multiple fibre orientations: What can we gain? NeuroImage 34:144-155 doi: <https://doi.org/10.1016/j.neuroimage.2006.09.018>

Camden C, Meziane S, Maltais D, Cantin N, Brossard-Racine M, Berbari J, Couture M (2019) Research and knowledge transfer priorities in developmental coordination disorder: Results from consultations with multiple stakeholders. Health Expect doi: 10.1111/hex.12947

Esser P, Weedon BD, Meaney A, et al. (2019) Brain plasticity and motor skill competence development in adolescents with poor motor skill acquisition and performance: Trial Protocol for a controlled experimental paradigm. Open Science Framework doi: 10.17605/OSF.IO/W8GJ6

Jenkinson M, Beckmann CF, Behrens TEJ, Woolrich MW, Smith SM (2012) FSL. NeuroImage 62:782-790 doi: <https://doi.org/10.1016/j.neuroimage.2011.09.015>

Kemp S, Dawes, H, Morris, M (2013) INSPORT “Promoting Social Inclusion through Sport “ Good Practices : Oxford Brookes “Have a GO” Sports day. In: Study Report to Sport England

Salimi-Khorshidi G, Douaud G, Beckmann CF, Glasser MF, Griffanti L, Smith SM (2014) Automatic denoising of functional MRI data: Combining independent component analysis and hierarchical fusion of classifiers. NeuroImage 90:449-468 doi: <https://doi.org/10.1016/j.neuroimage.2013.11.046>

Smith SM, Jenkinson M, Johansen-Berg H, et al. (2006) Tract-based spatial statistics: Voxelwise analysis of multi-subject diffusion data. NeuroImage 31:1487-1505 doi: <https://doi.org/10.1016/j.neuroimage.2006.02.024>

Winkler AM, Ridgway GR, Webster MA, Smith SM, Nichols TE (2014) Permutation inference for the general linear model. NeuroImage 92:381-397 doi: <https://doi.org/10.1016/j.neuroimage.2014.01.060>

Woolrich MW, Ripley BD, Brady M, Smith SM (2001) Temporal Autocorrelation in Univariate Linear Modeling of FMRI Data. NeuroImage 14:1370-1386 doi: <https://doi.org/10.1006/nimg.2001.0931>
